# Growth charts for children aged 6–17 years in Shanxi, China: comparison with other cities of China and WHO

**DOI:** 10.1186/s12887-024-04905-w

**Published:** 2024-07-02

**Authors:** Guoqiang Hou, Kunkun Li, Wenjun Cao, Mengmeng Tao, Wei Tian, Ruimin Chang

**Affiliations:** 1 Changzhi Maternal and Child Care Hospital, Changzhi, Shanxi 046000 China; 2 School of Public Health, Shanxi Medical University, Taiyuan, Shanxi 030000 China; 3 Department of Preventive Medicine, Changzhi Medical College, No.161, East Jiefang Road, Changzhi, Shanxi 046000 China

**Keywords:** Children, Body mass index, LMS method, Growth reference

## Abstract

**Background:**

Growth charts are an important method for evaluating a child’s health, growth, and nutritional status. It is essential to monitor the growth of children and adolescents using growth charts.

**Objectives:**

To present body mass index (BMI)-for-age references reflecting children’s growth in Shanxi. We also compare our new data with growth references of other cities of China and World Health Organization (WHO) growth standards.

**Methods:**

A stratified cluster random sampling method was used to recruit 5461 children and adolescents aged 6–17 years. Height and weight were measured and BMI was calculated. The LMS method was used to calculate the percentile values of body mass index by sex and age. Smoothed BMI-for-age growth curves were presented for both sexes and compared with reference data from other cities of China and WHO.

**Results:**

BMI centiles increased with age but with different patterns in both boys and girls. The centile curves from the 3rd to the 50th had a slight increase, while a sharp increase was seen from 11 to 17 years in boys and from 6 to 14 years in girls in the higher centiles. In comparison with other cities of China, the values for the 50th percentile are higher than those reported for children from China 2009, Shanghai, Changsha and China 2010 in both sexes. In comparison with WHO growth references, Chinese girls and boys had higher values in all percentiles, whereas curves of girls look roughly the same. The medians for BMI in Shanxi increase linearly from 6 to 17 years in boys.

**Conclusions:**

The BMI percentiles of children aged 6–17 years in Shanxi differed significantly from the growth reference curves of other cities of China and WHO. Recommending the provision of BMI reference curves for local children and adolescents to assess their growth and development and monitor their nutritional status.

Early detection of overweight and obesity in children provides a scientific basis for the prevention and control of overweight and obesity in children.

## What is known about this subject

●The constructed growth reference curves now are mainly for the southern cities and some big cities such as Beijing and Shanghai. However, there is little information about cities with a medium level of economic development in our country’s north.

## What this study adds

● BMI reference curves reflecting the growth of children aged 6-17 in Shanxi was presented.

● We compare our new data with the growth charts of more regions in China than just the WHO.

## Introduction

Influenced by genetics, biology, psychosocial factors and health behaviors, overweight and obesity in childhood is a complex public health problem that has become a global epidemic in developed countries, leading to a range of diseases that contribute to increased morbidity and premature mortality [1]. Based on data compiled by the Global Burden of Disease Collaboration for 2000 and 2013, we have estimated that by 2025, approximately 268 million children aged 5–17 are likely to be overweight, including 91 million obese children, assuming that no policy interventions have proven effective at changing current trends [2]. In less than a generation, the prevalence of overweight and obesity among children worldwide has increased dramatically. Prevalence data from 52 studies conducted in 16 of the 28 States in India show that the prevalence of overweight/obesity in children and adolescents increased from 16.3% in 2001–2005 to 19.3% after 2010 [3]. Over the past four decades, the rates of overweight and obesity among children and adolescents in China have increased rapidly, with the latest national prevalence estimates from 2015 to 2019 as follows: 6.8% overweight and 3.6% obesity in children under 6 years of age; Children and adolescents aged 6 to 17 years have an overweight rate of 11.1% and an obesity rate of 7.9% [4]. Therefore, special interventions and strategies monitoring and tracking the level of nutritional status in accordance to children’s healthy growth is of extreme importance.

Body mass index (BMI) is an important indicator reflecting the body shape and nutritional status of the human body. With the increasing prevalence of childhood obesity worldwide, more and more scholars have studied and advocated the use of BMI as an indicator for monitoring and screening of childhood overweight and obesity [5]. In 1990, Prof. Cole first applied the LMS method to construct the BMI growth curves for children and adolescents in the United Kingdom [6], and after that, many countries and regions around the world successively developed their own growth curves for children and adolescents [7–13]. Based on the data obtained from the “*2005 Physical Development Survey of children under 7 years old in nine cities of China*” and the “*2005 Survey on Physical Fitness and Health of Students in China*”, China also constructed the BMI percentile curves of Chinese children aged 0–18 years by using the LMS method in 2009 [14]. However, China is a vast country with unequal economic development between various areas, which makes the physical development of children and adolescents uneven among regions, and corresponding BMI percentile reference values should be formulated for different regions. Pan et al. [15] compared the distribution of body mass index by age in four cities in eastern China. Zhou et al. [16] used the LMS method to establish the growth curves of body mass index for 3- to 11-year-old children in Changsha. Qiu et al. [17] developed BMI percentile curves by age, sex and urban–rural regions for Beijing children and compared the results with Chinese national data and international references. Yang et al. [18] constructed the BMI percentile curves of Shanghai children and adolescents aged 7–18 years with LMS method, which can directly reflect the growth and development of Shanghai children and adolescents, and pointed out that their BMI is higher than the national level. The growth reference curve constructed by the above research is mainly for the southern cities and some big cities such as Beijing and Shanghai. However, there is little information about cities with a medium level of economic development in our country’s north.

Therefore, the main purpose of this study was to present body mass index (BMI) percentile curves in the northern region with medium level of economic development in order to monitor the growth of its children and adolescents. We also compare our new data with growth references of other cities of China and WHO growth standards.

### Participants and methods

#### Participants

From October to November 2021, a total of 12 schools, including 4 primary schools, 4 junior high schools and 4 high schools, with 1 to 3 classes in each grade, with a total of 5,610 students, were selected by stratified cluster random sampling method in Yanhu District and Wanrong County, Yuncheng City, Shanxi Province. Students with other age groups and data deletions, students with chronic diseases such as digestive diseases, metabolic diseases, cardiovascular diseases, and endocrine system diseases were excluded, students with physical disabilities and developmental abnormalities were excluded. BMI data outside the M ± 5S range for each age group were excluded to prevent extreme data from interfering with the curve fitting in the reference population, and 5461 participants aged 6 to 17 years were finally included.

### Anthropometric measures

The height was measured using designated height Measuring instruments (Wuxi Weighing Instrument Factory Co., Ltd. TZG type). The participants stood barefoot, with the back to the column on the height meter base, with the torso naturally straight, the head upright and the eyes looking straight ahead. The upper limbs hung naturally and the legs were straight; the heels were together and the toes were about 60° apart; the heels, the sacrum and the scapulae are in contact with the column, forming a “three points one line” stance. The body mass was measured using designated electronic weighing scales (Shanghai Taizhiheng Electronic Weighing Instrument Technology Co., Ltd. G&G tc-200 k type). The participants wore light clothes and bare feet and stood naturally in the middle of the weighing tray, keeping the body stable. Before measurement, height measuring instruments and electronic scales were tested and calibrated to ensure their accuracy and stability; before the formal investigation, the quality control personnel conducted unified and standardized training for all survey personnel, including the use of measuring tools and the process of collecting survey information. At the same time, every day in a randomized manner in accordance with the proportion of 5% of the subjects on the height, weight quality control, testing and detection error, height allowable error 0.5 cm, weight allowable error 0.1 kg, if the daily incidence rate of > 5%, should be studied for reasons and methods of improvement; if the incidence rate of > 10%, then the data of the day is invalid, to be re-tested.

### Smoothing methods

The LMS method [19] assumes that the outcome variable has a normal distribution after a Box-Cox power transformation is applied. Three smoothing and specific curves for each age were obtained via penalized maximum likelihood, namely: M (median), L (Box-Cox transformation) and S (coefficient of variation). The equation to derive the centiles is the following:

$$C_{100\alpha}\left(\mathrm t\right)={M\left(\text{t}\right)\lbrack1+L\left(\text{t}\right)S\left(\text{t}\right){\text{Z}}_\alpha\rbrack}^{1/L(\text{t})}$$

where ${Z}_{\alpha }$ is the normal equivalent deviate for tail area α, ${C}_{100\alpha }$(t) is the centile corresponding to ${Z}_{\alpha }$ Equivalent degrees of freedom (edf) for *L*(t), *M*(t) and *S*(t) measure the complexity of each fitted curve. The appropriate number of degrees of freedom was selected on the basis of the deviance, Q-tests and worm plots following the suggestions of Royston and Wright [20], van Buuren and Fredricks [21] and Pan and Cole [22, 23]. The 3rd, 5th, 10th, 15th, 25th, 50th, 75th, 85th, 90th, 95th and 97th percentiles were chosen as age- and sex-specific reference values.

### Statistical analysis

Epi Data 3.1 software was used to establish the database and double entry was made by two people. After the entry was completed, the variables were logically corrected to exclude outliers. The age was calculated to the precise day by subtracting the date of birth from the date of examination. SPSS version 26.0 software was used for data processing and statistical analysis. Quantitative information was described using means and standard deviations. The t-test was used for Height, Weight and BMI variables to compare the differences between the two groups. Categorical and hierarchical data were described as frequencies and percentages, and the chi-square test was used for Age categories and BMI categories variables to compare the differences between groups. The LMS Chartmaker Light, version 2.54 was used to develop age-related centiles for boys and girls and to smooth and fit the model. After developing age and sex specific centiles, the percentile curve graphs were plotted by using SAS 9.4 software. For the purpose of comparison, we plotted the 50th percentiles of Chinese children by using SAS 9.4 software against the corresponding values in the WHO [13]. We also compared the reference curves presented in this study with the references of other cities of China: China 2009 [14], China 2010 [24], Shanghai [18], Changsha [16]. *P* < 0.05 was considered a statistically significant difference.

### Ethics approval

This study involving human participants was in accordance with the ethical standards of the institutional and national research committee and with the 1964 Helsinki Declaration and its later amendments or comparable ethical standards. The Human Investigation Committee (IRB) of Changzhi Medical College approved this study (No. RT2023049).

## Results

There were 5461 children and adolescents, including 2750 boys, accounting for 50.4%, and 2711 girls, accounting for 49.6% in this study. Its average age was 11.6 (3.3) years.

The age was categorized into two groups, 6–11 years and 12–17 years, which showed no statistically significant differences (${x}^{2}$= 11.760, *P* = 0.382). The mean height, weight, and BMI of the participants were 149.6 (16.3) cm, 46.6 (16.9) kg, and 20.09 (4.1) kg/m^2^, respectively, and the differences were statistically significant (*P* < 0.05). The average height, weight and BMI of boys were higher than those of girls. The number of overweight and obese boys was 574 and 510, respectively, and the number of overweight and obese girls was 498 and 417, respectively; The prevalence of overweight and obesity in boys were 20.9% and 18.5%, and the prevalence of overweight and obesity in girls were 18.4% and 15.4%, respectively. The number of overweight and obese boys was significantly higher than that in girls, with statistically significant differences in obesity between boys and girls (*P* < 0.05). See Table 1.

**Table 1:** Basic characteristics of children and adolescents aged 6–17 in Shanxi province

variables	Boys(*n* = 2750)	Girls(*n* = 2711)	Total(*n* = 5461)	t/${x}^{2}$-value	*P*-value
Age categories				11.760	0.382
6–11 years	1334(48.5)	1327(48.9)	2661(48.7)		
12–17 years	1416(51.5)	1384(51.1)	2800(51.3)		
Height	151.5(17.9)	147.8(14.3)	149.6(16.3)	8.409	< 0.001^*^
Weight	48.3(18.6)	44.9(14.9)	46.6(16.9)	7.569	< 0.001^*^
BMI	20.2(4.3)	19.9(3.9)	20.09(4.1)	2.677	< 0.001^*^
BMI categories				19.322	< 0.001^*^
Normality	1666(60.6)	1796(66.2)	3462(63.4)		
Overweight	574(20.9)	498(18.4)	1072(19.6)		
Obesity	510(18.5)	417(15.4)	927(17.0)		

^*^p < 0.05 strong evidence against null hypothesis of no difference

The BMI centile values of children and adolescents were shown in Tables 2 and 3. The BMI 50th centile in boys increased gradually from 6 to 12 years (16.25–19.64 kg/ m^2^) and then increased substantially from 13–16 years (20.23–20.88 kg/ m^2^) and finally increased slowly. See Table 2. A different pattern was seen in girls, with a gradual and constant increase in BMI from 6 to 9 years (16.05–17.28 kg/m^2^) and a notable increase from 10 to 15 years (17.96–21.88 kg/m^2^) and a slight increase from 16 to 17 years (22.37–22.77 kg/m^2^). See Table 3.

**Table 2:** Age-specific L, M, S and smoothed percentile values of BMI for boys

Ages (year)	*L*	*M*	*S*	Body mass index (kg/m^2^)										
				3%	5%	10%	15%	25%	50%	75%	85%	90%	95%	97%
6	-2.31	16.25	0.11	13.80	14.04	14.45	14.74	15.21	16.25	17.56	18.42	19.10	20.27	21.18
7	-2.07	16.84	0.12	13.93	14.21	14.68	15.03	15.59	16.84	18.45	19.54	20.40	21.92	23.12
8	-1.88	17.40	0.14	14.08	14.40	14.93	15.33	15.97	17.40	19.27	20.55	21.56	23.38	24.83
9	-1.73	17.98	0.15	14.32	14.67	15.26	15.69	16.40	17.98	20.07	21.50	22.64	24.69	26.33
10	-1.65	18.53	0.15	14.61	14.98	15.61	16.07	16.83	18.53	20.78	22.33	23.56	25.79	27.56
11	-1.63	19.08	0.16	14.97	15.36	16.01	16.50	17.29	19.08	21.45	23.09	24.40	26.76	28.65
12	-1.63	19.64	0.16	15.38	15.78	16.46	16.96	17.78	19.64	22.11	23.81	25.19	27.67	29.66
13	-1.66	20.23	0.16	15.85	16.26	16.96	17.47	18.32	20.23	22.79	24.56	25.99	28.59	30.70
14	-1.70	20.88	0.16	16.37	16.79	17.51	18.04	18.91	20.88	23.53	25.37	26.87	29.61	31.84
15	-1.73	21.55	0.16	16.91	17.35	18.08	18.63	19.52	21.55	24.30	26.22	27.79	30.66	33.03
16	-1.76	22.18	0.16	17.42	17.86	18.61	19.17	20.09	22.18	25.02	27.01	28.64	31.64	34.13
17	-1.79	22.76	0.16	17.87	18.33	19.10	19.67	20.61	22.76	25.67	27.73	29.42	32.55	35.15

**Table 3:** Age-specific L, M, S and smoothed percentile values of BMI for girls

Ages (years)	*L*	*M*	*S*	Body mass index (kg/m^2^)										
				3%	5%	10%	15%	25%	50%	75%	85%	90%	95%	97%
6	-2.51	16.05	0.10	13.74	13.97	14.35	14.63	15.07	16.05	17.30	18.13	18.77	19.90	20.78
7	-2.32	16.46	0.11	13.83	14.09	14.52	14.83	15.34	16.46	17.91	18.90	19.67	21.05	22.13
8	-2.16	16.78	0.12	13.88	14.16	14.63	14.97	15.53	16.78	18.41	19.52	20.41	22.00	23.27
9	-2.01	17.28	0.13	14.11	14.42	14.93	15.30	15.91	17.28	19.08	20.30	21.29	23.05	24.46
10	-1.88	17.96	0.14	14.53	14.86	15.41	15.82	16.48	17.96	19.90	21.23	22.29	24.18	25.68
11	-1.76	18.82	0.14	15.13	15.49	16.08	16.52	17.23	18.82	20.89	22.30	23.42	25.40	26.96
12	-1.66	19.71	0.14	15.78	16.16	16.80	17.27	18.03	19.71	21.90	23.37	24.52	26.56	28.14
13	-1.56	20.54	0.14	16.39	16.79	17.46	17.96	18.76	20.54	22.82	24.34	25.53	27.59	29.19
14	-1.47	21.27	0.14	16.91	17.34	18.05	18.57	19.41	21.27	23.63	25.19	26.41	28.50	30.10
15	-1.38	21.88	0.15	17.35	17.79	18.53	19.08	19.96	21.88	24.32	25.92	27.15	29.27	30.87
16	-1.30	22.37	0.15	17.67	18.13	18.90	19.47	20.38	22.37	24.87	26.49	27.75	29.87	31.47
17	-1.22	22.77	0.15	17.92	18.40	19.20	19.78	20.72	22.77	25.32	26.97	28.24	30.38	31.97

Comparison of the fitted values at the 50th percentile of BMI with the measured values at the 50th percentile in Table 4 showed that the differences between the measured values and the fitted values for all age groups were basically within the permissible errors of measurement, which showed that the results of this method were more in line with the reality and the fitting results were better. See Table 4.

**Table 4:** Comparison of the fitted and measured values of the 50th percentile of body mass index at different ages

Age (years)	Boys			Girls		
	Measured values	Fitted values	Differences	Measured values	Fitted values	Differences
6	16.06	16.25	-0.19	16.03	16.05	-0.02
7	16.72	16.84	-0.12	16.52	16.46	0.06
8	17.01	17.40	-0.39	16.45	16.78	-0.33
9	18.27	17.98	0.29	17.34	17.28	0.06
10	18.44	18.53	-0.09	17.85	17.96	-0.11
11	19.64	19.08	0.56	19.05	18.82	0.23
12	19.56	19.64	-0.08	19.54	19.71	-0.17
13	19.72	20.23	-0.51	20.47	20.54	-0.07
14	20.23	20.88	-0.65	20.88	21.27	-0.39
15	21.49	21.55	-0.06	22.48	21.88	0.6
16	22.12	22.18	-0.06	22.66	22.37	0.29
17	22.46	22.76	-0.3	22.00	22.77	-0.77

**Fig. 1:**
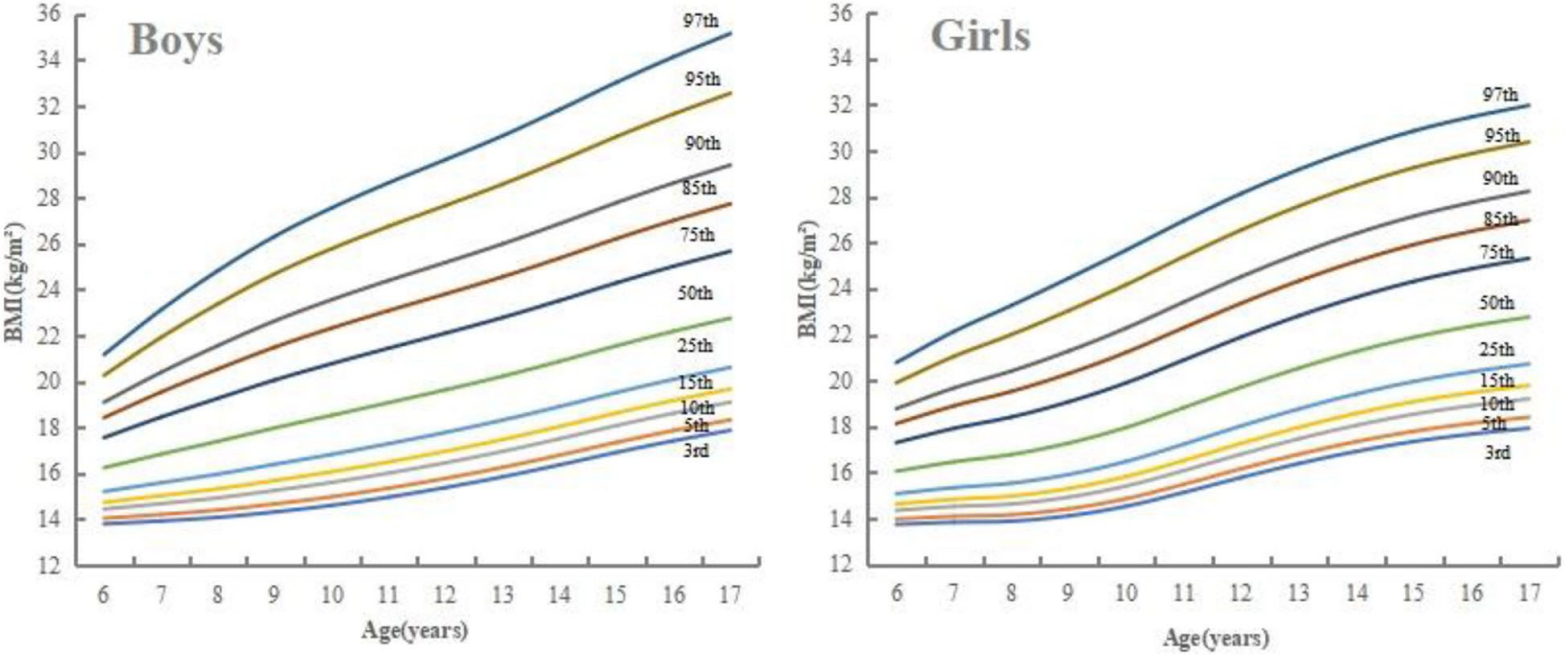
Smoothed reference curves for body mass index in 6 to 17 years-old boys and girls

Figure 1 showed the smoothened body mass index curves for boys and girls aged 6–17 years old in the study, using 3rd, 5th, 10th, 15th, 25th, 50th, 75th, 85th, 90th, 95th and 97th percentiles, respectively. Equivalent BMI percentile values were shown in Tables 2 and 3. In both boys and girls, BMI centiles increased with age but with different patterns. The centile curves from the 3rd to the 50th had a slight increase, while a sharp increase was seen from 11 to 17 years in boys and from 6 to 14 years in girls in the higher centiles.

**Fig. 2:**
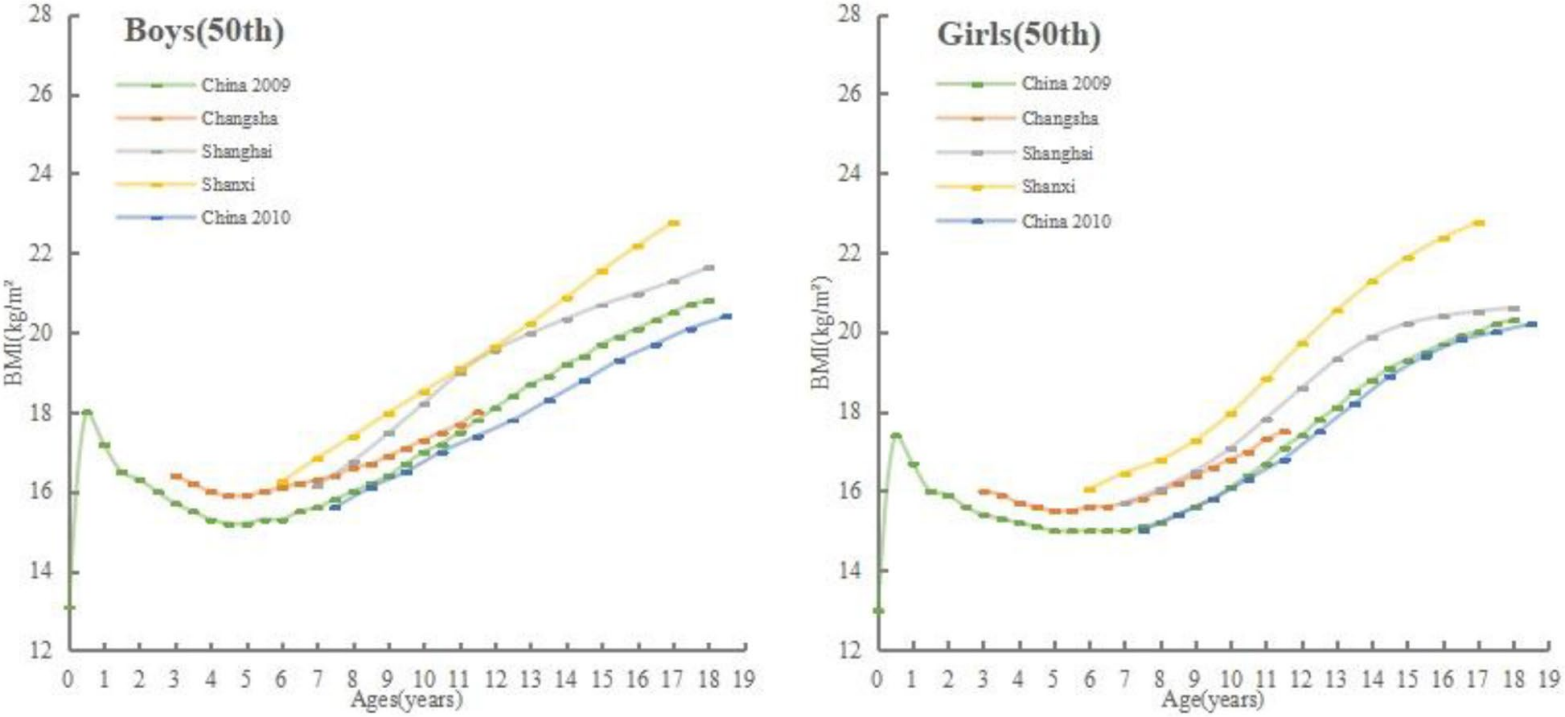
Comparison of the 50th percentile BMI curves of children in Shanxi with other cities of China

Figure 2 showed comparisons between the reference curves of the 50th percentiles of BMI for boys and girls s in Shanxi and the reference curves of other cities in China. In both sexes the reference values of the 50th percentiles in Shanxi were higher than those reported for children from China 2009, Shanghai, Changsha and China 2010. In comparison of boys, there were some intersections between the five curves, while in comparison of girls, there were also some intersections between the other four curves, except for the curve in the 50th percentile of girls in Shanxi Province. The BMI reference values for boys and girls aged 3–11 years in Changsha were larger than those in China 2009, and both curves show a decline followed by an increase in the 3–11 age group. In boys, the medians of BMI for boys in China 2009 and China 2010 increased linearly from 8 to 18 years of age, and the 50th percentile reference values for boys in China 2009 were greater than those for boys in China 2010. In contrast to the linear growth of the reference curve for boys aged 6–17 years in Shanxi Province, the curve for boys in Shanghai grew rapidly at ages 7–11 years and slowly at ages 12–18 years. In girls, the difference between the reference values for Chinese 2009 and Chinese 2010 children aged 8–18 was relatively small, with both reference curves showing rapid growth between the ages of 8 and 15 and a slowing down of growth after the age of 15. Compared with the reference values for children in Shanxi, the reference values for children aged 7–17 years in Shanghai were all smaller than those in Shanxi, and both the reference curves for children in Shanxi and Shanghai grew rapidly at ages 7–13 years, whereas the reference curves for children in Shanxi continue to grow rapidly at ages 13–17 years, while the reference curves for children in Shanghai flatten out.

**Fig. 3:**
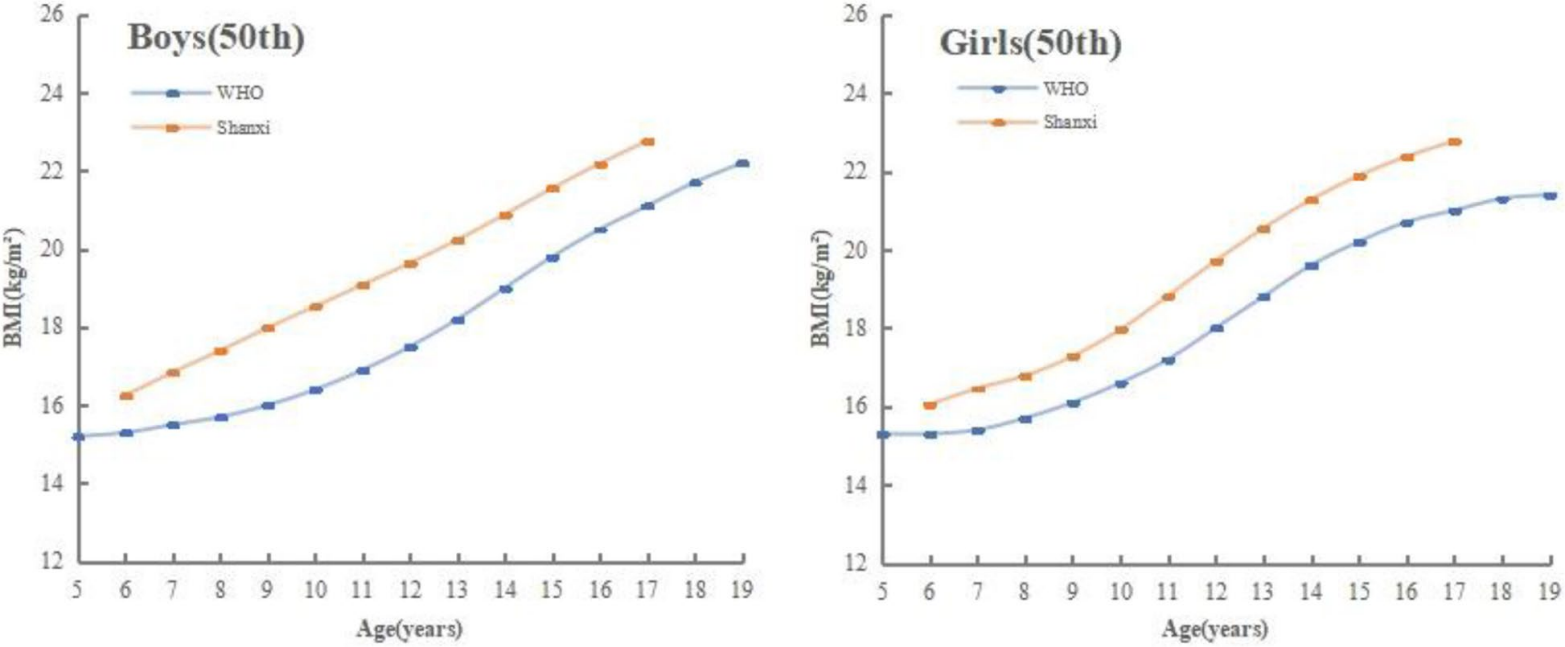
Comparison of the 50th percentile BMI curvesof children in Shanxi with WHO

Figure 3 showed comparisons between the reference curves of the 50th percentiles of BMI for boys and girls in Shanxi and the reference curves of WHO. Compared to the WHO reference values, the girls and boys in Shanxi had higher reference values in all percentiles. The medians for BMI in Shanxi increased linearly from 6 to 17 years in boys, whereas the growth trend of the reference curve for the 50th percentile of BMI in girls was roughly the same as that of the WHO, both showing a gradual and sustained increase in BMI before the age of 9, a notable increase from 10 to 15 years old and a slight increase after the age of 16.

## Discussion

Cross sectional reference percentiles curves based on data collected in 2021 for body mass index for boys and girls in Shanxi, China aged 6–17 years old are presented. This is the first known attempt to describe the distribution of BMI among children aged 6–17 years with a moderate level of economic development.

The results of this study showed different patterns of BMI percentile curves between Shanxi boys and girls and documented that Shanxi boys were fatter than Shanxi girls, which is consistent with previous findings [15, 17, 25]. Sex differences for growth and development patterns could account for this phenomenon. It is commonly recognized that boys gain greater fat free mass and skeletal mass, whereas girls tend to have more fat mass at all ages [26, 27]. The main discrepancy in the BMI curves of boys and girls after 14 years is the rapidly elevated fat free mass in boys compared to girls during post-puberty [26]. On the other hand, the traditional concepts of ‘boys are superior to girls’ in China and the prevalence of preference for thin body image among adolescent girls may also partly explain this phenomenon [25].

The BMI reference curves may vary depending on factors such as geography, culture, level of economic development, ethnicity, and nutritional status [28–31]. In this study, the BMI reference values for children and adolescents in Shanxi increased with age, which is consistent with many studies at home and abroad [14, 16–18]. Our study showed that the BMI reference values for boys and girls aged 6–17 years in Shanxi were higher than the Chinese standard [14, 24], which may be related to the higher prevalence of overweight and obesity among children and adolescents in Shanxi in recent years [32].

In this study, the BMI reference values for children in Shanxi were greater than those in Shanghai and Changsha, which may be related to the large differences in geography, dietary habits, economy and medical level between the north and south of China. Previous studies have shown that the population in northern China is fatter than that in the south, which is attributed to the longer winters and colder climate in the northern region, where the local residents tend to exercise less, coupled with high-calorie dietary habits and excessive intake of large amounts of carbohydrates without proper control, which may lead to excess energy and ultimately convert to fat accumulation in the body, increasing the risk of obesity [15, 33, 34]. Another reason why the reference values of BMI for children in Shanxi are greater than those in Shanghai and Changsha may be the COVID-19 epidemic in 2021 and the resulting lack of physical activity, which is undoubtedly an unprecedented health crisis and challenge for children and adolescents. Long term home restrictions can bring about health problems, including physical inactivity, sedentary behavior, weakened immunity, weight gain and more [35].

The studies showed that children living at high altitude and exposed to chronic environmental stress had lower height, weight and BMI values compared to children living at sea level [36, 37], which is inconsistent with the results of this study that BMI reference values of children living at high altitude in Shanxi province were higher than those in Shanghai, and consistent with the results of this study that BMI reference values of children living at high altitude in Changsha were lower than those in Shanghai.

In addition, the higher BMI reference values for children in Shanghai than in Changsha may be related to the superior economic strength and development potential of Shanghai compared to Changsha. One of the consequences of economic growth is changes in diet and lifestyle. Nowadays, children in Shanghai spend more time watching TV [25] or playing computer games than engaging in outdoor activities. Many Shanghai urban dwellers have moved into high-rise housing and now rely on subways, taxis and buses instead of bicycles for transportation, which reduces physical exercise. Western-style food is increasingly available. Western-style fast food restaurants (QSR) such as KFC and McDonald have grown rapidly in China’s major cities since the first KFC restaurant opened in Beijing in 1987 [38]. This was closely followed by the opening of the first KFC in Shanghai on December 8, 1989, which was deeply loved by young people in the local area. This disproportionately increased the intake of dietary fat for children in Shanghai [39].

International comparisons for the BMI percentile curves help to clarify features of each population and the BMI percentile curves can be used to assess the health of the population. Compared to the WHO references values, the 50th percentile reference curves for both boys and girls in Shanxi were higher than the WHO, consistent with the research results of Esmaili et al. [40]. BMI is mostly used to identify obesity in adults. In children, it is used as a screening tool for possible obesity and hence as a measure of adiposity. Shanxi children had higher BMI values in comparison with the WHO child growth standards. These higher values may suggest that Shanxi children are at a high risk of childhood obesity.

The WHO child growth standards represent ideal child growth and it is ideal for all children globally, for growth under optimum conditions. This is in line with the Sustainable Development Goals and the Nutrition Global targets. However, global conditions under which children grow vary immensely with inequalities in income, access to diet and cultural diversity. And the study by Anesu et al. [41] shows that the WHO growth charts do not apply to the Zimbabwean region of Africa. Thus, the development of regional growth references is more likely to reflect the environmental, geographical and sometimes socio-economic influences on child growth. But it cannot be generalized, and the study by El Shafie et al. [42] shows that the reference curves for the growth of children and adolescents aged 5–19 years established in Egypt were very close to the WHO. This mean that WHO growth charts may be appropriate for monitoring growth and nutritional status on Egyptian children. In summary, international standards are not fully applicable to all countries and regions of the world.

Limitations and prospects of the present study should be mentioned. First, only 5874 children and adolescents from a city in Shanxi Province were selected for this study, which sample size may be small and lack of representativeness, but the sample size calculated by Cochran formula is sufficient. Despite the sufficient sample, the extrapolation of the results still requires multi-center, representative studies to verify and improve due to the influence of environmental, dietary, economic factors. Second, this study was based on the cross-sectional investigation of the growth and development of children and adolescents between the ages of 6 and 17 years, with no information on longitudinal growth. Further prospective cohort studies will be required to evaluate the impact of cohort effects on growth patterns in children. In addition, children and adolescents outside the age of 6–17 years old were not included in the study. Future studies may consider incorporating some indicators such as the incidence rate of common diseases, tracking the risk factors of adult onset, and predicting adult onset, so as to prevent them early. Finally, in view of the continuous increase in the overweight and obesity rates of children and adolescents in Shanxi Province, it is recommended to further improve the “student-family-school-medical care” four-in-one obesity prevention and treatment model.

## Conclusion

In conclusion, we provided BMI percentile reference curves for children aged 6–17 years in Shanxi, and found that the curves were significantly different from growth reference curves in other Chinese cities and the World Health Organization. Recommending the provision of BMI reference curves for local children and adolescents to assess their growth and development and monitor their nutritional status.

Early detection of overweight and obesity in children provides a scientific basis for the prevention and control of overweight and obesity in children.

## Data Availability

The datasets used and/or analysed during the current study available from the corresponding author on reasonable request.

## Abbreviations

BMI: Body mass index
WHO: World Health Organization
